# Characterization of zebrafish rod and cone photoresponses

**DOI:** 10.21203/rs.3.rs-5984163/v1

**Published:** 2025-03-12

**Authors:** Shinya Sato, Vladimir Kefalov

**Affiliations:** University of California, Irvine

**Keywords:** photoreceptor cells, phototransduction, zebrafish, ex vivo electroretinography

## Abstract

Zebrafish is a popular species widely used in vision research. The zebrafish retina has one rod and four cone subtypes (UV-, blue-, green-, and red-sensitive) with 40%-rod 60%-cone ratio, making it suitable for comparable studies of rods and cones in health and disease. However, the basic photoresponse properties of the four zebrafish cone subtypes have not been described yet. Here, we established a method for collecting flash photoresponses from zebrafish rods and cones by recording membrane current with a suction electrode. Photoreceptor subtypes could be distinguished based on their characteristic morphology and spectral sensitivity. Rods showed 40–220-fold higher photosensitivity than cones. In the four cone subtypes, green-sensitive cones showed the highest sensitivity, 5.5-fold higher than that of red cones. Unexpectedly, rods produced smaller flash responses than cones despite their larger outer segments. Dim flash response analysis showed the quickest response kinetics in blue- and red-sensitive cones, with responses about 2-fold faster than the responses of UV- and green-sensitive cones, and 6.6-fold faster than the rod responses. We also obtained pharmacologically isolated photoreceptor voltage responses (a-wave) from isolated zebrafish retinas using *ex vivo* electroretinography (ERG). Dim flashes evoked rod-only responses, while bright flashes evoked two-component responses with a slow rod component and a fast cone component. Red- and green-sensitive cones were the dominant sources of the overall cone response. These studies provide a foundation for the use of zebrafish rods and cones to study the fundamental mechanisms that modulate the function of vertebrate photoreceptors in health and disease.

## Introduction

Zebrafish is commonly used in life science research including fundamental studies of rod and cone photoreceptor cells. For example, recent studies have explored the following topics: cell fate specification^1^, mRNA and protein expression profiles^2,3^, mitochondrial homeostasis^4^, circadian regulation^5–8^, regeneration from injury^9–12^, visual cycle^13–16^, visual pigments^17,18^, and live imaging of retinogenesis^19^ and photoresponses^20^. Compared to the nocturnal mouse retina which is rod-dominant (97% rods^21^) and has only green- and UV-sensitive cone subtypes, diurnal zebrafish has cone-dominant retinas at larval stage (92% cones^22^) and ~ 60%-cone retinas at 3 to 6 months adult stage^23^, with four cone subtypes (UV, blue, green, and red). Thus, zebrafish studies can expand our understanding of cone photoreceptor function.

Electrophysiological photoresponses of zebrafish rods and cones have been described in previous studies, mainly by electroretinography (ERG) recordings. *In vivo* ERG responses have been recorded by placing an electrode just above the cornea of an anesthetized zebrafish larva^24–27^, or by inserting an electrode into the eye of an anesthetized adult fish^28^. *Ex vivo* ERGs have been recorded also by inserting an glass pipette electrode into an isolated larval eyeball^5,29^, or by placing the electrode inside the adult eyecup^29,30^. In some of these ERG studies, the photoreceptor response is obtained by blocking the b-wave pharmacologically^5,27,29,30^. Single-cell recordings have been reported for rods^31^, and each cone subtype: UV^32^, blue^33^, green^34,35^, and red cones^13,36^. However, a complete set of photoresponse characterization from the one rod and four cone classes in the zebrafish retina has not been described yet.

Here, we characterize the flash response properties of rods and cones in adult zebrafish retinas obtained by single-cell suction and *ex vivo* ERG recordings. Using suction recordings, we collected flash response families and determined spectral sensitivity, intensity-response relationships, and response kinetics from the five subtypes of zebrafish photoreceptor cells. Using *ex vivo* ERG recordings, we found that rod and cone mixed ERG a-wave is observed in bright light responses. These data provide a solid foundation for future applications of zebrafish in ophthalmology and vision science.

## Results

### Zebrafish rod and cone cellular morphology and spectral sensitivity.

**Figure 1:**
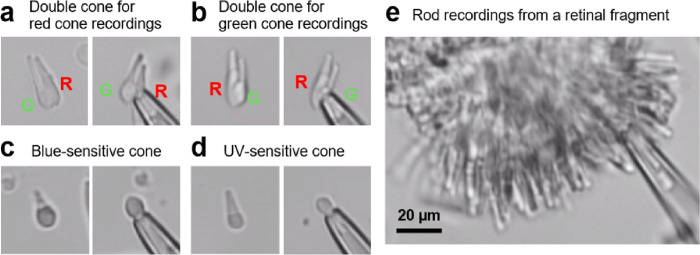
Microscopic images of dissociated zebrafish photoreceptor cells and a retinal fragment. The right picture in each panel shows a cell after being drawn into the recording pipette. (a and b) A double cone used for recordings from a red-sensitive cone (a), or a green-sensitive cone (b). R: a red cone, G: a green cone. (c and d) A single cone identified as a blue-sensitive cone (c) or an UV-sensitive cone (d). (e) A retinal fragment used for rod recordings.

First, we sought to establish the single-cell suction recording method from the five different subtypes of zebrafish photoreceptor cells. Fish were kept in darkness overnight, and retinas were dissected out under dim red and infrared illumination. For cone recordings, the retina was chopped into small pieces by a razor blade, and cells were dissociated by pipetting them in a plastic tube and then transferred into the perfusion chamber. Under a microscope with infrared illumination, we found double cones and single cones on the bottom of the chamber (Fig. 1). For single cones, an outer segment could be easily drawn into the recording pipette. However, for double cones, this was technically challenging because the two outer segments are physically too close to isolate one of them. Therefore, the ellipsoid part of a cone was drawn to obtain inverted current responses, which were re-inverted in the later data analysis. After cell dissociation, many cones had damaged appearance characterized by rough surface texture. Those damaged cones produced small sluggish responses, or no response at all. Therefore, recordings were performed only from cones with smooth surfaces.

**Figure 2:**
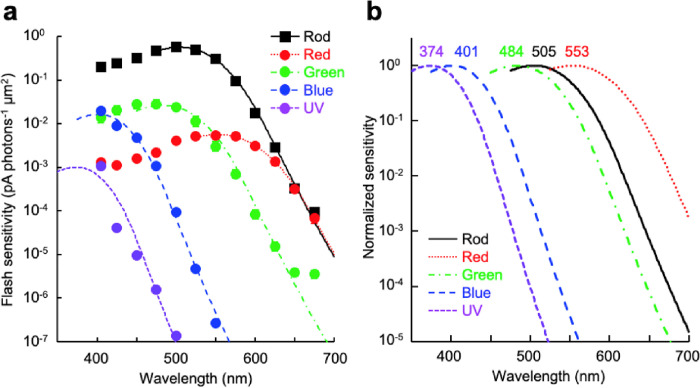
(a) Spectral sensitivity plots. Sensitivity data were fitted with the Govardovskii nomogram for the α-band of visual pigment absorption using parameters described in Table 1. Data are mean ± SEM, but error bars are too small to be visible for most of the plots. (b) Normalized fitting curves with estimated λ_max_ values.

Double cones are known to be composed of red-sensitive (or long-wavelength sensitive) and green-sensitive (middle-wavelength sensitive) cones, and single cones are blue-sensitive (short-wavelength sensitive 2) or UV-sensitive (short-wavelength sensitive 1)^37^. We confirmed this by spectral sensitivity analysis. The λ_max_ of the long-double cone (also known as principal member), which has a larger ellipsoid, was estimated at 553 ± 2 nm (Fig. 2, Table 1), corresponding to the reported value for red-sensitive cones (565 nm^17^ or 549 nm^38^). The λ_max_ of the short-double cone (accessory member) was at 484 ± 2 nm (Fig. 2, Table 1), which is almost identical to the published value for green-sensitive cones (482 nm^17,38^). Intriguingly, the sensitivity of green-sensitive cones at 675 nm was 10-fold higher than the value predicted from fitting the spectral template. Similar observation was reported for UV-sensitive cones^32^, which is explained by the ectopically expressed green- or red-sensitive pigment, or both, in a UV cone. However, our data could not be fitted well with a red and green mixed spectrum (Supplementary Fig. S1). Blue- and UV-sensitive single cones are known as long and short single cones, respectively^3^.

After cell dissociation, they were hard to distinguish based on morphology but could be easily identified by their characteristic spectral sensitivity (Fig. 2, Table 1): cones showing estimated λ_max_ at 401 ± 2 nm were identified as blue-sensitive, and cones showing estimated λ_max_ at 374 ± 1 nm were identified as UV-sensitive. These values do not agree well with the reported λ_max_ of blue-sensitive cones (411 nm^17^ or 425 nm^38^) and UV-sensitive cones (361 nm^17^ or 342 nm^38^), but correspond to them. Therefore, in the following experiments, we determined the cell type of single cones based on their spectral sensitivity. In practice, cones were identified by comparing their responses to 405 nm and 450 nm flashes with the same photon density.

For rod recordings, the chopped retinal fragments were directly transferred into the chamber. The cell dissociation step was skipped to reduce possible mechanical damage. The λ_max_ of rods was estimated at 505 ± 1 nm (Fig. 2, Table 1), which is almost identical to the reported values for rods (503 nm^17^ or 502 nm^38^). Taken together, these results demonstrate successful establishment of the single-cell suction recording method from the five photoreceptor cell types in the zebrafish retina and characterization of their specific action spectra. Hereafter, we characterize the sensitivity and response kinetics of these photoreceptor types.

### Intensity-response relationships of zebrafish rods and cones.

**Figure 3:**
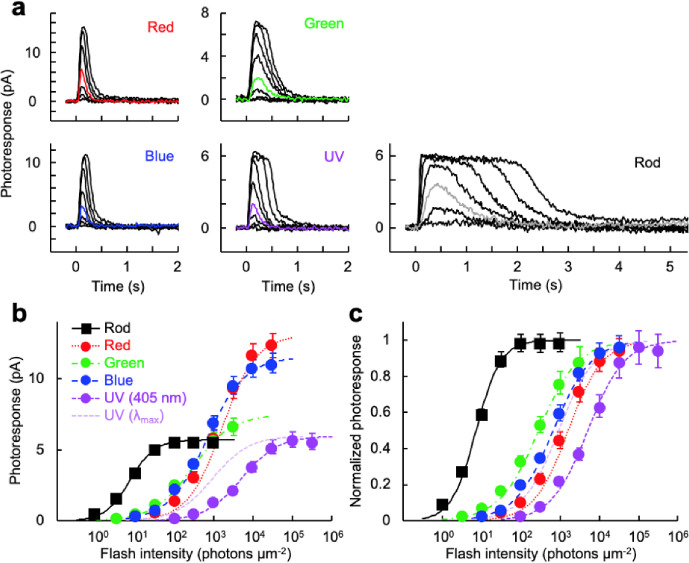
Representative response families and intensity-response relationships obtained by single-cell suction recordings. (a) Response families from the five zebrafish photoreceptor subtypes. Flash wavelength and intensity: red-sensitive cones (Red), 575 nm, 32 to 320,000 photons μm^−2^, red trace: 1000 photons μm^−2^; green-sensitive cones (Green), 500 nm, 3.2 to 10,000 photons μm^−2^, green trace: 100 photons μm^−2^; blue-sensitive cones (Blue), 405 nm, 10 to 32,000 photons μm^−2^, blue trace: 320 photons μm^−2^; UV-sensitive cones (UV), 405 nm, 100 to 320,000 photons μm^−2^, violet trace: 3,200 photons μm^−2^; rods, 500 nm, 1 to 1,000 photons μm^−2^, gray trace: 10 photons μm^−2^. (b, c) Intensity-response relationships of zebrafish photoreceptor cells in absolute values (b) and in normalized values (c). The light purple curve is obtained after adjustment for the 5.5-fold higher sensitivity at λ_max_ compared to 405 nm. Plots are fitted with Naka-Rushton equation described in Methods and parameters shown in Table 2. Data are mean ± SEM. Numbers of data points are shown in Table 2.

Flash response families (Fig. 3a) were obtained from dark-adapted rods and cones to analyze their maximum response amplitude and sensitivity (Fig. 3b, Table 2). The wavelength of stimulation for each cell type was selected based on their respective λ_max_. But UV-sensitive cones were stimulated with 405 nm violet light because our equipment did not have a UV light source. Based on the reported absorption spectra of live cells obtained by microspectrophotometry^17^, the absorption of the UV cone pigment at λ_max_ is 5.5-fold higher than that at 405 nm. Therefore, we added curves (Fig. 3b and c, light purple) and values (Table 2) adjusted for that 5.5-fold difference. Despite their larger outer segment size (Fig. 1), rods showed the smallest response amplitude among the five photoreceptor subtypes (Fig. 3a, Table 2, *R*_*max*_). As expected, rod sensitivity was 40–220-fold higher than that of cones based on their half-saturating flash intensity values (Fig. 3b, Table 2, *I*_*1/2*_). The kinetics of the flash response was clearly slower in rods than in cones, as quantitatively compared in the dim flash response analysis below. Among the four cone subtypes, red- and blue-sensitive cones showed larger responses than green- and UV-sensitive cones (Fig. 3b and Table 2, *R*_*max*_). Intriguingly, the *I*_*1/2*_ sensitivity of green-sensitive cones was 5.5-fold higher than that of their fellow red-sensitive cones (Table 2). The *I*_*1/2*_ sensitivity of UV-sensitive cones to 405 nm light was the lowest among the four cone subtypes. However, after the aforementioned adjustment to their λ_max_, the sensitivity of UV cones was comparable to that of blue-sensitive cones and in between green- and red-sensitive cones (Fig. 3b, light purple curve). The photoresponses of red- and blue-sensitive cones were faster than those of green- and UV-sensitive cones, as compared quantitatively in the next section.

### Dim flash response kinetics of zebrafish rods and cones.

**Figure 4:**
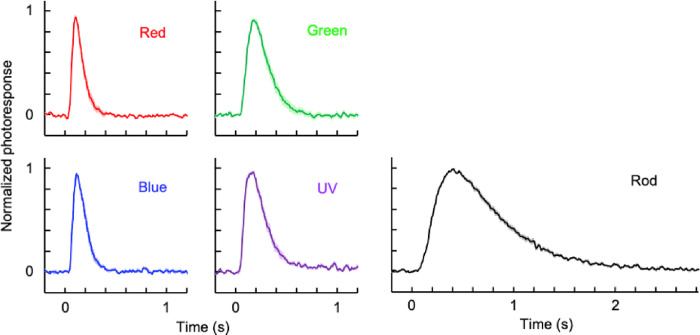
Averaged dim flash responses of zebrafish photoreceptor cells. Data are mean ± SEM. The numbers of cells for each averaged trace are shown in Table 3.

Dim flash responses were collected from the five subtypes of photoreceptor cells to compare their response kinetics (Fig. 4). A dim flash is defined as a flash intensity in the linear region of the intensity-response relationships (Fig. 4), which is typically intensity producing a response with amplitude < 30% of *R*_*max*_. Based on the appearance of normalized and averaged dim flash responses, the kinetics of the responses was in the following order: red- and blue-sensitive cones > green- and UV-sensitive cones > rods. For quantitative comparison, three kinetic parameters — time to peak (*T*_*p*_), integration time (*T*_*int*_), and recovery time constant (*τ*_*rec*_) — were obtained for each cell type (Table 3). Integration time is the area of the normalized response, and recovery time constant is the time constant of the exponential fit to the recovery phase of the response. Based on *T*_*int*_, the responses of red- and blue-sensitive cones were approximately 2-fold faster than the responses of green- and UV-sensitive cones, and 6.6-fold faster than the responses of rods. Analysis of *T*_*p*_ and *τ*_*rec*_ showed a similar trend.

### Ex vivo ERG a-wave recordings from the zebrafish retina.

**Figure 5:**
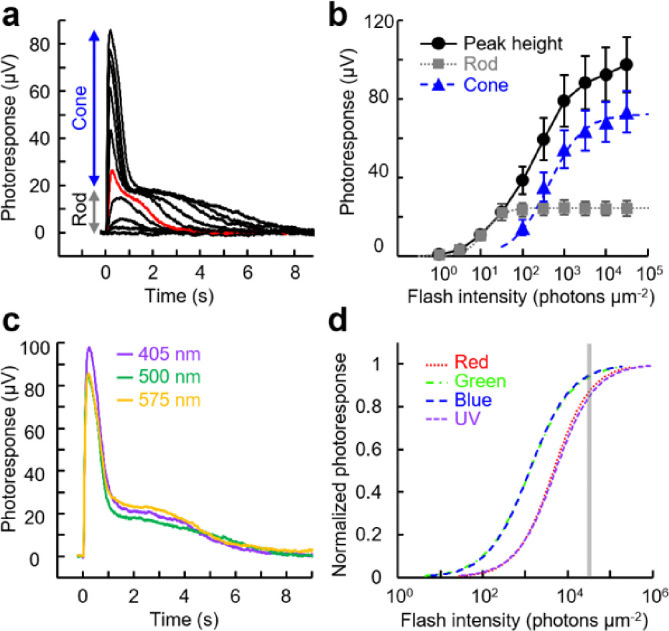
(a) Representative flash response family from zebrafish rods and cones obtained by *ex vivo* ERG recordings. The ERG a-wave was inverted to positive direction for consistency with the single-cell recordings. Flash wavelength and intensity: 500 nm, 1 to 32,000 photons μm^−2^. Red trace was obtained at 100 photons μm^−2^. (b) Intensity-response relationships (●) and estimated rod (■) and cone (▲) components of the overall response. Rod and cone plots were fitted with Naka-rushton function with *R*_*max*_ = 25 μV, *I*_*1/2*_ = 13 photons μm^−2^, and *n* = 1.9 for rod data, and with *R*_*max*_ = 72 μV, *I*_*1/2*_ = 425 photons μm^−2^, and *n* = 1.0 for cones. Plots are mean ± SEM (n = 7). (c) Responses obtained with 405 nm (violet), 500 nm (green), and 575 nm (orange) flash at 32,000 photons μm^−2^. (d) Estimated intensity-response relationships of the four cone subtypes when stimulated with 405 nm flashes, based on their spectral sensitivity (Fig. 2) and intensity-response relationships obtained with near λ_max_ flashes (Fig. 3). A grey line indicates 32,000 photons μm^−2^.

Our group previously developed an *ex vivo* ERG adaptor for mouse retinas^39^. When used with inhibitors to block signals from downstream neurons and Müller cells (see Methods for details), the adaptor gives the photoreceptor component of the transretinal voltage response, as reported in mouse and human retina studies^39–41^. Here, we collected *ex vivo* ERG flash responses from the zebrafish retina (Fig. 5a). Two component flash responses were observed with bright flashes. The slow component detected with low intensities was identified as the rod component, and the fast component observed with 100 photons μm^−2^ and brighter flashes was identified as the cone component. This threshold intensity of cones is broadly consistent with the intensity-response relationships obtained by single-cell suction method (Fig. 4). *R*_*max*_ and *I*_*1/2*_ were estimated for rod and cone components by fitting estimated amplitude plots with the Naka-Rushton function. Cone *R*_*max*_ was 72 ± 10 μV (n = 7, mean ± SEM), which was 2.9-fold larger than the corresponding rod *R*_*max*_ (25 ± 4 μV). Rod *I*_*1/2*_ was 13 ± 2 photons μm^−2^, which was about twice the value obtained from single-cell suction recordings (7.3, Table 2). This difference could be caused by absorption or scattering of the test flashes in the acrylic *ex vivo* ERG adaptor. Cone *I*_*1/2*_ was 425 ± 76 photons μm^−2^, some 33-fold larger than rod *I*_*1/2*_. This is consistent with the 40-fold difference between the sensitivities of green cones and rods observed in the single-cell suction analysis (*I*_*1/2*_ of 290 and 7.3 photons μm^−2^, respectively, Table 2). Among responses to 405 nm, 500 nm, and 575 nm flashes at the same 32,000 photons μm^−2^ intensity, 405 nm flashes gave the largest cone response (Fig. 5c). This is because all cone subtypes respond to 405 nm violet light as shown in their spectral sensitivity plots (Fig. 2; perhaps counterintuitively, sensitivities to 405 nm light are comparable in UV- and red-sensitive cones), and 32,000 photons μm^−2^ flash is expected to produce saturating or semi-saturating responses from all four cone subtypes (Fig. 5d). Response amplitude to a 575 nm flash, to which blue- and UV-sensitive cones have virtually no sensitivity (Fig. 2), was still 80% of the largest 405 nm flash response, showing that red- and green-sensitive cones are the two dominant sources of the cone component. This observation is consistent with the population ratio of zebrafish cones: red- and green-sensitive cones are roughly twice as numerous as blue- and UV-sensitive cones^42^. Together, these results demonstrate that the rod and cone responses can be isolated from whole retina *ex vivo* ERG recordings.

**Figure 6:**
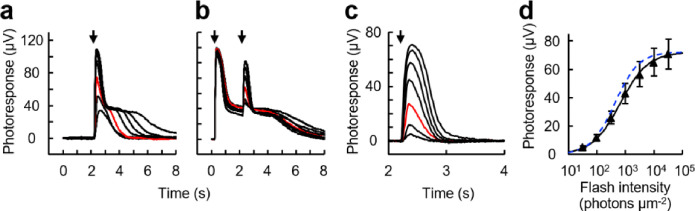
Isolation of cone responses by *ex vivo* ERG recordings. (a) Control response family. Flash was given one time at 2.2 s (arrow). Flash wavelength: 500 nm. Intensity: 32 to 32,000 photons μm^−2^. Red trace: 320 photons μm^−2^. (b) Responses to paired flashes. Flashes were given two times at 0.2 and 2.2 s (arrows). First flash: 500 nm, 32,000 photons μm^−2^. Second flash: same as in (a). (c) Isolated cone responses obtained by clipping 2–4 s in (b) with subsequent baseline adjustment. (d) Intensity-response relationship of cone responses. Plots were fitted with Naka-rushton function with *R*_*max*_ = 72 μV, *I*_*1/2*_ = 661 photons μm^−2^, and *n* = 0.89. Plots are mean ± SEM (n = 6). The fitting curve in Fig. 5b is shown in blue for comparison to demonstrate the slight suppression of the cone responses by the first flash.

We also established a protocol for isolating the cone component of the *ex vivo* ERG a-wave using a pair of 500 nm flashes (Fig. 6). A bright first flash was given to saturate rods for several seconds, and a second flash was given 2 seconds later to obtain a cone response (Fig. 6b). A response family was isolated from the paired flash responses (Fig. 6c), and the cone *I*_*1/2*_ was estimated to be 661 ± 75 photons μm^−2^ (n = 6, mean ± SEM) by fitting the intensity-response relationship (Fig. 6d). This value was 1.6-fold higher than the cone *I*_*1/2*_ estimated from single flash responses (Fig. 5b, 425 ± 76 photons μm^−2^). We tried to isolate rod component by subtracting the cone component from the overall rod-cone mixed responses (Fig. 6a). However, a small cone response-like bumps were left on the rod saturating plateau (Supplementary Fig. S2). This suggests that the cone responses elicited by the second flash were a little smaller than the cone responses elicited by an identical single flash. Together with the slightly higher *I*_*1/2*_ value, this result indicates that cones were desensitized to a detectable degree by the conditioning first flash.

## Discussion

In this study, we have established single-cell suction recordings from the five photoreceptor subtypes in the zebrafish retina (Figs. 1 and 2, Table 1) and collected basic response waveforms and parameters from them (Figs. 3 and 4, Tables 2 and 3). We also demonstrated a method to collect and isolate the rod and cone derived responses using the *ex vivo* ERG adaptor (Figs. 5 and 6). We hope that these results will facilitate and boost the use of zebrafish in vision research.

Sensitivities varied largely among the four cone subtypes (Figs. 1 and 3, Table 2), with green-sensitive cones showing 5.5-fold higher sensitivity than the fellow red-sensitive cones. A very similar 5.9-fold difference has also been reported for goldfish red- and green-sensitive cones^43^. In contrast, the three cone subtypes in primate *Macaca fascicularis* show comparable sensitivity^44^. What is the molecular mechanism of the large sensitivity difference in fish cones? Based on the previous molecular analyses in zebrafish, phototransduction components of green and red cones are almost identical except for their opsin genes: opn1lw1 in red cones and opn1mw1, opn1mw2, opn1mw3, or opn1mw4 in green cones^3^. Therefore, the lower red cone sensitivity could be ascribed to its visual pigment. Microspectrophotometry analysis of goldfish cones has shown the presence of ~ 30% apo-opsin in red-sensitive L-cones, in contrast to only ~ 3% in green-sensitive M-cones and negligible fraction in blue-sensitive S-cones^43^. Apo-opsin is formed in cones even in darkness by spontaneous chromophore dissociation from opsin protein^45^. In salamander red cones, exogenously added 11-*cis* retinal, which binds to apo-opsin to form stable visual pigment, effectively increases sensitivity by two-fold^45^. However, this was not observed prominently in goldfish red-sensitive cones^43^. We will examine the effect of exogenous 11-*cis* retinal on zebrafish cones in the future. Another possible source of desensitization from pigments is their spontaneous light-independent activity (dark noise). In salamander cones, the frequency of spontaneous pigment activation rate was estimated to be 600 s^−1^ in L-cones and < 2 s^−1^ in S-cones. Corresponding to this large dark noise in L-cones, the estimated single photon response of an L-cone is 0.04 pA, which is 5.8-fold smaller than that from an S-cone (0.23 pA)^46^. In contrast, such frequent thermal activation is not observed in the human red cone pigment (~ 8.8 s^−1^) when expressed in mouse rods ectopically^47^. A systematic study of noise properties in zebrafish cone photoreceptors will be needed to clarify the molecular mechanism of sensitivity differences among cone subtypes.

Perhaps surprisingly, we found that the amplitude of the rod response was smaller than that of the cones (Fig. 3, Table 2 *R*_*max*_), even though rods have the largest outer segments among the five photoreceptor subtypes (Fig. 1). The rod amplitude, 5.7 pA, is consistent with previously published zebrafish rod data (5.5 pA)^31^. Reported rod amplitudes measured by the suction method are varied among fish species: 12.4 pA in skate^48^, 9.2 pA in goldfish^43^, 36.7 pA in striped bass^49^, and 3.5 pA^50^ or 4.9 pA^51^ in carp. zebrafish and carp belong to the same *Cyprinidae* family, hence small rod response might be a common feature in this family. Consistently, the rod component in the *ex vivo* ERG a-wave was only about 1/3 of the corresponding cone component (Fig. 5). The adult zebrafish retina has 40%-rod 60%-cone ratio^23^. Therefore, the smaller rod a-wave does not originate from the smaller number of rods but is due largely to the smaller responses of individual rods. What is the molecular mechanism of the smaller responses in zebrafish rods? One possibility is the circadian regulation of the response amplitude. Rods mediate nighttime vision due to their 40–220-fold higher sensitivity compared to cones (Fig. 3, Table 2, *I*_*1/2*_). However, all results presented in this study were obtained only in daytime when rod function is supposed to be suppressed. Fish rods and cones show dynamic day-night regulation in multiple biological processes including rod-cone gap junction^52^, retinomotor movement^53^, and disappearance of ERG b- and d-waves after the disorganization of cone ribbon synapses in midnight^5^. These regulations work to increase photosensitivity of the retina in nighttime, probability of photon capture in rod outer segments, and suppress cone function, respectively. The rod response might be boosted specifically in nighttime to enhance night vision. To address this question directly, we are currently collecting day-night comparison data from rods and cones. Another possible reason for the relatively small rod responses is that the perfusion solution used in this study might not be particularly suitable for rods because of the different metabolism in rods and cones^54–56^. This point could be clarified by *in vivo* ERG recordings of pharmacologically isolated photoreceptor a-waves from adult zebrafish.

Dim flash response kinetics were faster in red- and blue-sensitive cones than in UV- and green-sensitive cones. The response shutoff process in red cones is thought to be accelerated by the pigment-derived noise discussed above. This noise would work as equivalent background light in darkness to drive the calcium-feedback mechanisms, which would upregulate the response shutoff through mechanisms mediated by recoverin/S-modulin and guanylate cyclase activating proteins (GCAPs)^57–59^. Another possible factor modulating the response kinetics could be the different expression patterns of phototransduction proteins among the four cone subtypes. At mRNA level, *grk7b* is enriched specifically in UV-sensitive cones among the four photoreceptor Grk subtypes, and *arr3b* is enriched in UV- and blue-sensitive cones among the four photoreceptor arrestin subtypes^2^. However, these expression patterns do not provide a simple explanation for the quicker response in blue-sensitive cones over UV- and green-sensitive cones. To resolve this issue, more quantitative insights will be required, particularly at the protein level. For example, Zang and colleagues have shown corresponding but different circadian regulations of mRNA and protein for Grk7a and Arr3a in adult zebrafish eyeballs^6^.

The *ex vivo* ERG method provided pharmacologically isolated a-waves from rods and cones. This approach is advantageous to the single-cell suction method in several ways. One is the higher signal-to-noise ratio of transretinal recordings. Typical signal-to-noise ratio was 30–40 in the raw trace of our *ex vivo* ERG recordings. In contrast, it was only 5–10 in the single-cell suction recordings, requiring denoise by averaging many responses. This point is particularly important if analyzing small responses from degenerating retinas from disease model animals. For example, we previously obtained transretinal voltage signals from severely degenerated *Rpe65*^−/−^*Grk1*^−/−^ mouse retinas from which suction recordings were not possible^60^. Secondly, for transretinal recordings, the retina is kept mostly intact. This allows us to study not only a-wave responses from photoreceptor cells but also b-wave responses, produced mainly by ON bipolar cells, as well as the slow PIII component from Müller glial cells, by comparing photoresponses in the presence of different set of inhibitors^39,61^. ERG recordings from the intact retina also enable the study of the interaction between cones and Müller cells in the retinoid metabolism^62^. Thirdly, our *ex vivo* ERG adaptor could be set up in a commercial ERG system as well^61^, and recently, a detailed method for recording from the zebrafish retina with the Diagnosys Espion ERG system was published from another group^63^.

Compared to the rod-dominant mouse retina (97% rods^21^), zebrafish has cone-dominant retina at larval stage (92% cones^22^) and ~ 60%-cone retina at 3 to 6 months adult stage^23^. Many human eye diseases including age-related macular degeneration and Leber congenital amaurosis type 2 lead to cone degeneration. In addition, in retinitis pigmentosa, preservation of the remaining cone function after rod death is of great interest in ophthalmology research. Understanding the physiology of cones in health and disease will advance our understanding of these diseases. Studies of cone-enriched zebrafish retinas in combination with genetic manipulations, single-cell genomics, proteomics, functional imaging, and electrophysiology hold exciting potential for addressing many fundamental questions in visual sciences.

## Methods

### Chemicals

DL-2-amino-4-phosphonobutyric acid (DLAP4) was obtained from Tocris Bioscience (Bristol, UK). All other chemical reagents were obtained from Sigma-Aldrich (Saint Louis, MO).

### Animals

All experiments were performed in accordance with the NIH Guide for the Care and Use of Laboratory Animals and the Association for Research in Vision and Ophthalmology Statement for the Use of Animals in Ophthalmic and Vision Research, and were approved by the Institutional Animal Care and Use Committee of UC Irvine. Authors complied with the ARRIVE guidelines. Fish were purchased from the zebrafish International Resource Center at University of Oregon and kept at room temperature (22°C) under scheduled 14 hours light-10 hours dark illumination cycle. Fish were fed commercial fish foods (Micro Pellets, Hikari, Hyogo, Japan) two or three times a day. Prior to the experiments, each zebrafish was transferred into a 250 mL plastic beaker with approximately 150 mL water and kept in darkness overnight for complete dark adaptation of photoreceptor cells. Fish were euthanized by rapid cooling in ice-cold water followed by decapitation.

### Single cell suction recordings

Eyes were enucleated, and the cornea and lens were removed using a pair of micro scissors and fine forceps under a stereomicroscope with an infrared illuminator and infrared scopes. The retinas were peeled off in Ringer’s solution (104 mM NaCl, 2 mM KCl, 1.6 mM MgCl_2_, 1.5 mM CaCl_2_, 30 mM NaHCO_3_, and 10 mM glucose, pH 7.3 by bubbling with 95%O_2_/5%CO_2_) and chopped into small pieces randomly with a razor blade. For rod recordings, retinal fragments were directly transferred into the perfusion chamber. For cone recordings, cones were dissociated from the retina by pipetting the retinal fragments in a plastic tube with a wide-bore glass pipette, typically for 15 times, and then immediately transferred into the recording chamber. Perfusion was temporarily halted for 10 min until cells were settled down on the bottom of the chamber. During a recording session, cells were kept under constant perfusion of Ringer’s solution (1.2 mL/min, 22°C) and imaged under infrared illumination. A single rod or cone was drawn into the suction pipette for flash response recordings. Whenever possible, only the outer segment part of the cell was drawn. But outer segments of red- and green-sensitive cones were found to be too close to draw only one of them. In that case, the ellipsoid part of the inner segment was drawn to obtain inverted current responses, which were re-inverted digitally in the subsequent data analysis. Both action and reference electrodes were filled with electrode solution in which 30 mM NaHCO_3_ in Ringer’s solution was substituted to NaCl and HEPES (129 mM NaCl, 2 mM KCl, 1.6 mM MgCl_2_, 1.5 mM CaCl_2_, 10 mM HEPES, and 10 mM glucose, pH 7.3 by NaOH). The cell was exposed to flash stimuli delivered from a custom-made light-emitting diode (LED) system^64^. Light intensity was calibrated with an optometer (350 Linear/Log optometer, UDT Instruments, San Diego, CA). Membrane current signals were amplified with an amplifier (Axopatch 200B, Molecular devices, San Jose, CA), low pass filtered (8pole Bessel at 30 Hz; Model 3382, KrohnHite, Brockton, MA) and imported to a computer through a digitizer (Digidata 1440A, Molecular Devices). Data analysis was done with Clampfit 10 (Molecular Devices). Other details are written and recorded in our previous video^65^.

When needed, the second eyecup from the fish was transferred to a 35 mm diameter culture dish with 2 mL of Ringer’s solution and stored at room temperature (22°C) in a dark box supplied with humidified 95%O_2_/5%CO_2_ gas until used for recordings.

Intensity-response relationships were fitted with Naka-Rushton hyperbolic function:

$$R=R_{max}\cdot I^{n}/\left(I^{n}+{I_{1/2}}^{n}\right)$$

where $R_{max}$ is maximum response amplitude (pA for here, μV for *ex vivo* ERG data), I is flash intensity (photons μm^−2^), n is the Hill coefficient, and $I_{1/2}$ is half-saturating flash intensity (photons μm^−2^)

The λ_max_ values for rods and cones (Table 1) were estimated by fitting the sensitivity plots (Fig. 2) with the Govardovskii nomogram for the α-band of visual pigment absorption^66^. Data points used for fitting were 405 to 500 nm for UV-sensitive cones, 405 to 550 nm for blue-sensitive cones, 450 to 650 nm for green-sensitive cones, 525 nm to 675 nm for red-sensitive cones, and 450 to 675 nm for rods.

### Ex vivo ERG

Eyes were collected as described above. The retinas were peeled off in Ringer’s solution supplemented with 20 μM DL-AP4 and 2 mM sodium aspartate^67^ to block the postsynaptic components of the photoresponse, and with 100 μM BaCl_2_ to suppress the slow glial PIII component^68^. Because aspartate deactivates glutamate input to both ionotropic and metabotropic receptors, DL-AP4 which blocks only the metabotropic receptor^30^ is probably not required. However, as DL-AP4 is usually used in our mouse studies, it was added in our early attempts of zebrafish recordings and was kept in the formula throughout this study for consistency. The same supplemented solution was used for perfusion. The retina was placed in an *ex vivo* ERG adaptor (Ocuscience, Henderson, NV), perfused with Ringer’s solution (1.2 mL/min, 22°C), and exposed to calibrated flash stimuli (see above). Trans-retinal voltage was amplified with a differential amplifier (DP-311A, Warner Instruments, Hamden, CT), low pass filtered (8pole Bessel at 30 Hz) and imported to a computer through a digitizer. Data analysis was done with Clampfit 10. Other details are written and recorded in our previous paper and video articles^39,61^.

## Figures and Tables

**Table 1:** Spectral sensitivity parameters (Mean ± SEM)

	λ_max_ (nm)	Estimated flash sensitivity (10^−3^ pA photons^−1^ μm^−2^)	n
Red-sensitive cones	553 ± 2	5.7 ± 0.5	5
Green-sensitive cones	484 ± 2	26 ± 7	5
Blue-sensitive cones	401 ± 1	16 ± 1	5
UV-sensitive cones	374 ± 1	1.0 ± 0.2	9
Rods	505 ± 1	570 ± 42	5

**Table 2:** Parameters obtained from intensity-response relationships.

	*R_max_* (PA)	*I_1/2_* (photons μm^−2^)	Hill coefficient	*S_f_* (pA photons^−1^ μm^−2^)	Fractional *S_f_* (10^−4^ photons^−1^ μm^−2^)	*n*
Red-sensitive cones	13 ± 1	1600 ± 400	1.00 ± 0.02	0.011 ± 0.002	8.2 ± 1.4	19
Green-sensitive cones	7.5 ± 0.7	290 ± 50	0.86 ± 0.02	0.038 ± 0.006	62 ± 12	22
Blue-sensitive cones	11.5 ± 0.7	660 ± 60	0.89 ± 0.01	0.020 ± 0.003	17 ± 2	18
UV-sensitive cones*	6.0 ± 0.6	5090 ± 460 (930 ± 80)	0.94 ± 0.02	0.0011 ± 0.0002 (0.0061 ± 0.0011)	2.1 ± 0.3 (12 ± 2)	18
Rods	5.7 ± 0.3	7.3 ± 0.6	1.39 ± 0.04	0.43 ± 0.04	760 ± 70	20

*S_f_* and Fractional *S_f_* were determined from cones whose photoresponse *R_max_* was 2.0 pA or larger. Fractional *S_f_* is obtained by dividing *S_f_* with *R_max_*.

* Sensitivity values for UV-sensitive cones are determined using violet light at 405 nm, which is 44 nm longer than their reported *λ*_max_ at 361 nm (Allison et al. 2004). Values in parentheses were obtained after the adjustment for the 5.5-fold higher sensitivity at *λ*_max_ compared to 405 nm.

**Table 3:** Response parameters from dim flash responses.

	*T_p_* (ms)	*T_int_* (ms)	*τ_rec_* (ms)	*n*
Red-sensitive cones	102 ± 5	120 ± 8	59 ± 5	19
Green-sensitive cones	180 ± 9	236 ± 16	125 ± 17	22
Blue-sensitive cones	119 ± 6	141 ± 7	63 ± 6	18
UV-sensitive cones	145 ± 8	241 ± 12	109 ± 11	14
Rods	413 ± 11	833 ± 26	486 ± 32	20

Data were determined from cones whose photoresponse *R_max_* was 2.0 pA or larger.

## Data Availability

The datasets generated during and/or analyzed during the current study are available from the corresponding author on reasonable request.
